# Fully Integrated Wearable Ultrasound System for Continuous Promotion of Wound Healing

**DOI:** 10.1002/advs.78619

**Published:** 2026-11-09

**Authors:** Faxing Zou, Yansen Wang, Yong Luo, Wenxuan Zhuang, Mingyu Zhu, Min Long, Qingzhou Liu, Tailin Xu

**Affiliations:** ^1^ School of Life and Health Technology Dongguan University of Technology Dongguan Guangdong China; ^2^ The Institute for Advanced Study (IAS) Shenzhen University Shenzhen Guangdong People's Republic of China; ^3^ Department of Medical and Health Materials SINOPEC Beijing Research Institute of Chemical Industry Beijing People's Republic of China; ^4^ Department of Endocrinology The First Affiliated Hospital (Southwest Hospital) of Army Medical University Chongqing People's Republic of China; ^5^ Guangdong Laboratory of Artificial Intelligence and Digital Economy (SZ) Shenzhen People's Republic of China

**Keywords:** antibacterial, chronic wound, promote healing, wearable ultrasound

## Abstract

Chronic wounds pose a significant global health burden due to their recalcitrance, high recurrence, and infection risks. Traditional treatments often lack consistent efficacy, multifunctionality, and dynamic adaptability to wound healing stages, prolonging patient distress and worsening outcomes. Here, we introduce an innovative intelligent Wearable Ultrasound System (iWUS) that seamlessly integrates a highly miniaturized ultrasound driving circuit, a drug‐loaded ultrasound patch for precisely controlled release of the antibacterial agent polyhexamethylene biguanide (PHMB), and a terminal for intuitive remote control, enabling noninvasive, on‐demand ultrasound stimulation to simultaneously promote tissue regeneration and combat infection. The iWUS combines biocompatibility, flexibility, and conformability for intelligent chronic wound management. It introduces an on‐demand dynamic therapy adapted to the wound microenvironment. In vitro assays showed sustained antibacterial action and enhanced cell migration. In vivo validation in a rat full‐thickness wound model demonstrated programmable bacterial inhibition, inflammation modulation, and improved collagen remodeling, significantly accelerating wound closure and promoting structural and functional skin regeneration. RNA sequencing analysis revealed that iWUS treatment upregulated pro‐healing genes (e.g., Bmp2, Egfr, Vegfd) while downregulating inflammatory pathways (e.g., NF‐κB, Tnf), elucidating its molecular mechanism in promoting tissue repair and resolving inflammation. The work paves the way for next‐generation wearable medical devices.

## Graphical abstract

A wearable ultrasound system integrating a drug‐loaded patch and a miniaturized driving circuit is presented to achieve noninvasive, on‐demand stimulation, enabling programmable antibacterial activity, inflammation modulation, and accelerated wound closure through enhanced cell migration and collagen remodeling. It introduces a novel strategy for chronic wound management and shows great significance in advancing wearable medical devices in therapeutic applications.

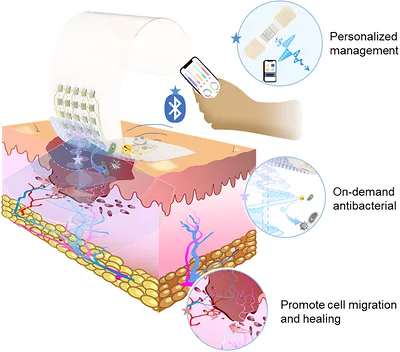

## 1 Introduction

As precision medicine gains momentum and the pursuit of high‐quality life intensifies, there is growing recognition that effective chronic wound management and the application of precision medicine are becoming critical to enhancing health outcomes and quality of life [1, 2, 3]. This awareness is driving scientific exploration toward novel disease treatment strategies [4, 5]. Chronic wounds, encompassing conditions such as diabetic foot ulcers, venous ulcers, and pressure injuries, inflict significant psychological and physical distress on patients due to their recalcitrant nature, high recurrence rates, and susceptibility to infection [6, 7, 8]. The persistent failure to heal is largely attributable to inappropriate and non‐sustained treatment strategies [9, 10, 11]. The propensity for recurrence stems from the lack of effective, portable wound management protocols, whereas infection susceptibility arises from the limited multifunctionality of therapeutic regimens. Recent strategies, such as hyperthermia‐enhanced immunoregulatory hydrogels for alleviating ROS and hypoxia, self‐cascading hydrogels integrating antibacterial activity with PAMP neutralization, and procyanidin/Fe_3_O_4_ hydrogels enhancing antioxidation and vascularization [12, 13, 14]. yet deficiencies persist in long‐lasting antibacterial efficacy, inflammation‐modulating capacity, and pro‐healing effects [12, 15, 16]. These interconnected challenges starkly illuminate the limitations of conventional therapies: their inability to dynamically respond to the spatiotemporal demands of the complex wound healing process (e.g., hemostasis, inflammation, proliferation, remodeling) or to achieve precision modulation based on the wound microenvironment. Although diverse therapeutic strategies have been developed for chronic wound management, their variable efficacy and insufficient therapeutic stability impede scientific progress [6, 17, 18]. Fundamentally, current approaches often focus on single mechanisms or static interventions, failing to adequately integrate responsive materials (e.g., programmable drug‐releasing matrices) with multifunctional synergy tailored to specific healing phases. Overcoming this bottleneck necessitates the development of next‐generation ‘smart’ wound management platforms [19, 20]. Such platforms must integrate dynamic therapeutic adaptation with patient compliance management, enabling engineered intervention within the wound microenvironment [21]. This integrated approach is essential to overcome the multifaceted barriers of inconsistent efficacy, recurrence, and infection, ultimately achieving predictable and durable healing outcomes [22, 23].

Ultrasound, a form of mechanical energy transmitted through sound waves, has seen expanding applications in biomedical engineering and clinical therapy in recent years [24, 25]. As research into its therapeutic and monitoring capabilities continues to grow, scientists are increasingly exploring its multifunctional potential, particularly in areas such as tissue repair, drug delivery, and real‐time imaging, where it demonstrates distinct advantages [26, 27, 28]. Numerous studies have confirmed that ultrasound offers several notable benefits over conventional treatment strategies. For instance, its non‐invasiveness helps reduce patient discomfort and infection risks, while its adjustable intensity and frequency allow it to meet varying demands across different treatment phases [11, 19, 29, 30, 31, 32]. Furthermore, the ongoing miniaturization of ultrasound devices supports the development of intelligent systems, enabling remote or at‐home use and enhancing practical therapeutic applicability [33, 34, 35]. These characteristics collectively establish ultrasound as an emerging and promising technology in the field of smart medicine. However, the reliance of current ultrasound‐driving apparatus on bulky equipment and specialized operators constrains its portability and long‐term usability [36, 37]. Recently, Zhou et al. developed a programmable, self‐adaptive ultrasonic wireless implant that delivers targeted, quantitative stimulation to the spinal cord based on graded pain levels for the management of chronic wound pain. While the system provides adaptive, wireless stimulation, it offers symptomatic relief rather than curative treatment of the underlying disease [11, 38]. Jian et al. combined a miniaturized wearable ultrasound patch (WUP) with an ultrasound‐responsive piezoelectric nanocomposite hydrogel, offering a clinically translatable strategy for personalized treatment of chronic diabetic wounds; however, it lacks on‐demand control and long‐acting antibacterial and pro‐healing strategies [39]. Su et al. reported a stretchable electronic facial mask for ultrasound‐facilitated transdermal delivery, which demonstrates exceptional biological compatibility and mechanical adaptability, enabling non‐invasive and controllable drug administration, representing a pioneering strategy for the treatment of chronic dermatological conditions [37]. Moreover, the emergence of novel strategies for chronic pain management expands the scope of implementation approaches and the therapeutic potential of wearable ultrasound systems [8, 40, 41]. Recent advances in our laboratory in ultrasound device miniaturization and driving circuitry have enabled the development of sustainable, portable therapies for chronic wounds [3, 42, 43, 44]. Together with the growing prevalence of intelligent interactive systems, these advances pave the way for wearable ultrasound systems dedicated to chronic wound treatment and management.

Here, we present a wearable ultrasound system for intelligent treatment and management of chronic wounds. The system comprises highly integrated components including an ultrasound driving circuit, a drug‐loaded ultrasound patch, and a terminal. We conducted in vitro biocompatibility, promotion of cell proliferation and migration, antibacterial assays, and in vivo experiments using Infected chronic wound skin defect model to evaluate its comprehensive performance. Furthermore, the RNA sequencing (RNAseq) was performed to explore and analyze its mechanism of promoting wound healing, especially in terms of collagen remodeling and inflammation regulation. These studies demonstrate the potential of this wearable platform for intelligent chronic wound treatment and remote management. Our approach offers a new pathway to shift chronic wound care from static intervention to dynamic on‐demand response and regulation, enabling personalized and intelligent healing support. Furthermore, it provides a feasible strategy for the development of next‐generation wearable medical devices.

## 2 Results and Discussion

### 2.1 Design of the Fully Integrated Wearable Ultrasound System (iWUS)

This platform is a wearable system designed for chronic wound management, enabling in situ multimodal combinatorial therapy at the wound site. Its core functions comprise the delivery of personalized wound‐care protocols, on‐demand controllable antimicrobial activity to suppress infection, and the effective promotion of cell migration to accelerate tissue healing (Figure 1a). The integrated design is envisaged to facilitate dynamic and precise modulation of the wound microenvironment, offering an innovative technological solution for the intelligent and efficient management of complex chronic wounds. The intelligent wound ultrasound system (iWUS) comprises three primary layers: a multimodal ultrasound array, a polyhexamethylene biguanide (PHMB)‐based dressing layer, and a flexible circuit layer (Figure 1b and Figure S1).

**FIGURE 1:**
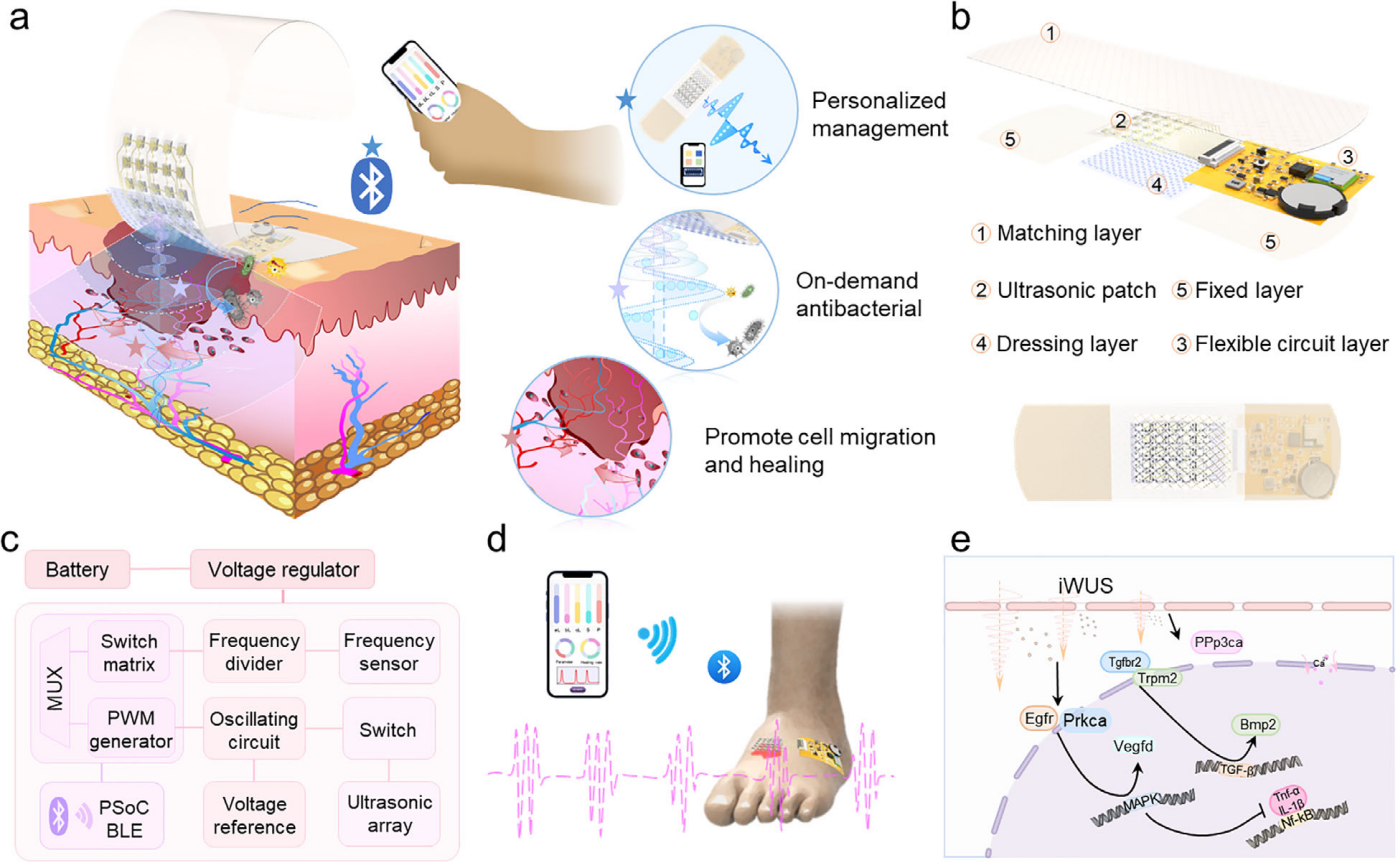
A fully integrated wearable ultrasound system (iWUS) for chronic wound management. (a) Schematic diagram of multifunctional iWUS treatment for chronic wound with promote cell migration and healing, on‐demand antibacterial drug release and antibacterial property, and personalized wound management; (b) Photograph and schematic of layered structure of iWUS that assembles the ultrasonic patch and dressing layer with matching layer and fixed layer into a compact electronic dressing housing; (c) Functional diagram of the system showing hardware blocks including transducer array, a control circuit and a battery. The control circuit is composed of oscillating module and frequency divider module for signal generation and amplification, and BLE module (Bluetooth Low Energy) for wireless transmission and control etc. (d) Schematic of a soft wearable system on a chronic nonhealing wound on a diabetic foot; e) Signal pathway diagram of the treatment process. (Created with PowerPoint).

The actuation of the ultrasound module generates localized mechanical and acoustic stimuli, which orchestrate two key therapeutic processes: they guide the directional migration of stem cells residing within the wound bed and provide spatiotemporal control over the release kinetics of the antimicrobial agent (PHMB) from the dressing layer. This integrated capability establishes a foundation for an intelligent terminal system designed to enable on‐demand, personalized wound‐management protocols. The precisely engineered circuit architecture‐integrating power supply, oscillation circuit, voltage regulation, and Bluetooth modules‐facilitates multifunctional operation, optimal energy management, and streamlined user interfacing. Key schematic implementations, including Bluetooth Low Energy (BLE) and Pulse Width Modulation (PWM) systems, are detailed in Figure 1c. The synergistic integration of mechanical flexibility, environmental adaptability, and embedded intelligence optimizes the device's clinical utility while enhancing patient compliance and therapeutic comfort [45, 46, 47]. These engineered characteristics further streamline human—computer interaction through intuitive interfaces and on‐demand response algorithms, thereby extending the operational scope across diverse medical scenarios—from point‐of‐care settings to personalized home‐based therapy—while maintaining robust treatment efficacy (Figure 1d). Following a series of systematic in vivo investigations, the proposed therapeutic mechanisms and associated pathways are illustrated in Figure 1e.

### 2.2 Characterization of iWUS System

The core part of an ultrasound system consists of an ultrasound drive circuit and an ultrasound patch (shown as Figure 2a,d). In addition, the portable button battery provides a convenient energy supply strategy for the ultrasound system, allowing it to perform wound treatment anytime and anywhere. The thinner thickness and flexible driving circuit board have laid the foundation for the implementation of portable medical devices (Figure 2b). The ultrasound‐driven system demonstrates sensitive start‐stop functionality (Figure 2c), reflecting its superior maneuverability. This flexible on‐demand actuation capability proves critical for portable medical devices, ensuring high responsiveness during dynamic usage contexts. Featuring purpose‐built modular architecture, the system enables instantaneous and precise ultrasound activation/termination control via intuitive remote operation through terminals, significantly enhancing both operational accessibility and efficiency. Furthermore, the intimate inter‐component assembly within the ultrasound patch constitutes an indispensable prerequisite for achieving efficient and reliable ultrasonic energy delivery. As shown in Figure 2d, high‐precision manufacturing enables robust integration of its multi‐layered architecture. Crucially, the incorporated stretchable metallic conductor ensures stable conductivity under deformation, while the elastic substrate provides both structural conformability to curvilinear surfaces and exceptional fatigue resistance (Figure 2e). This synergistic integration allows the patch to dynamically adapt to tissue morphological variations, simultaneously maintaining the same excellent mechanical properties as PDMS (Figure 2f). The Figure 2g shown the numerical simulation of the ultrasound field generated by the custom‐designed ultrasound stimulation during running, from which we can know that the ultrasound can penetrate depths exceeding 15 mm and is suitable for managing and treating various types of wounds on human skin. In addition, the ultrasound energy field of the system stimulates drug release through focused energy (as shown in Figure 2h, red dashed line), and its transverse ultrasound can promote drug diffusion and penetration (as shown in Figure 2h, yellow dashed line), with the therapeutic ultrasound parameters including a frequency of 1 MHz, an electrical output power of 5.4 W, a sound intensity of 1.7 W/cm^2^, a duty cycle of 50%, a pulse repetition frequency of 1.0 MHz, and a pulse length of 5 × 10^−^^7^ s. In the longitudinal cross‐sectional view in Figure 2i, the ultrasound energy focusing zone is distributed near the superficial surface, while the deeper region (indicated by the white dashed line in Figure 2i) forms a wave‐shaped ultrasound energy zone. Within this ultrasound energy field, cells migrate from high‐energy regions toward low‐energy regions [48]; during treatment, this substantially promotes the migration and diffusion of stem cells within the wound microenvironment (Figure 2j).

**FIGURE 2:**
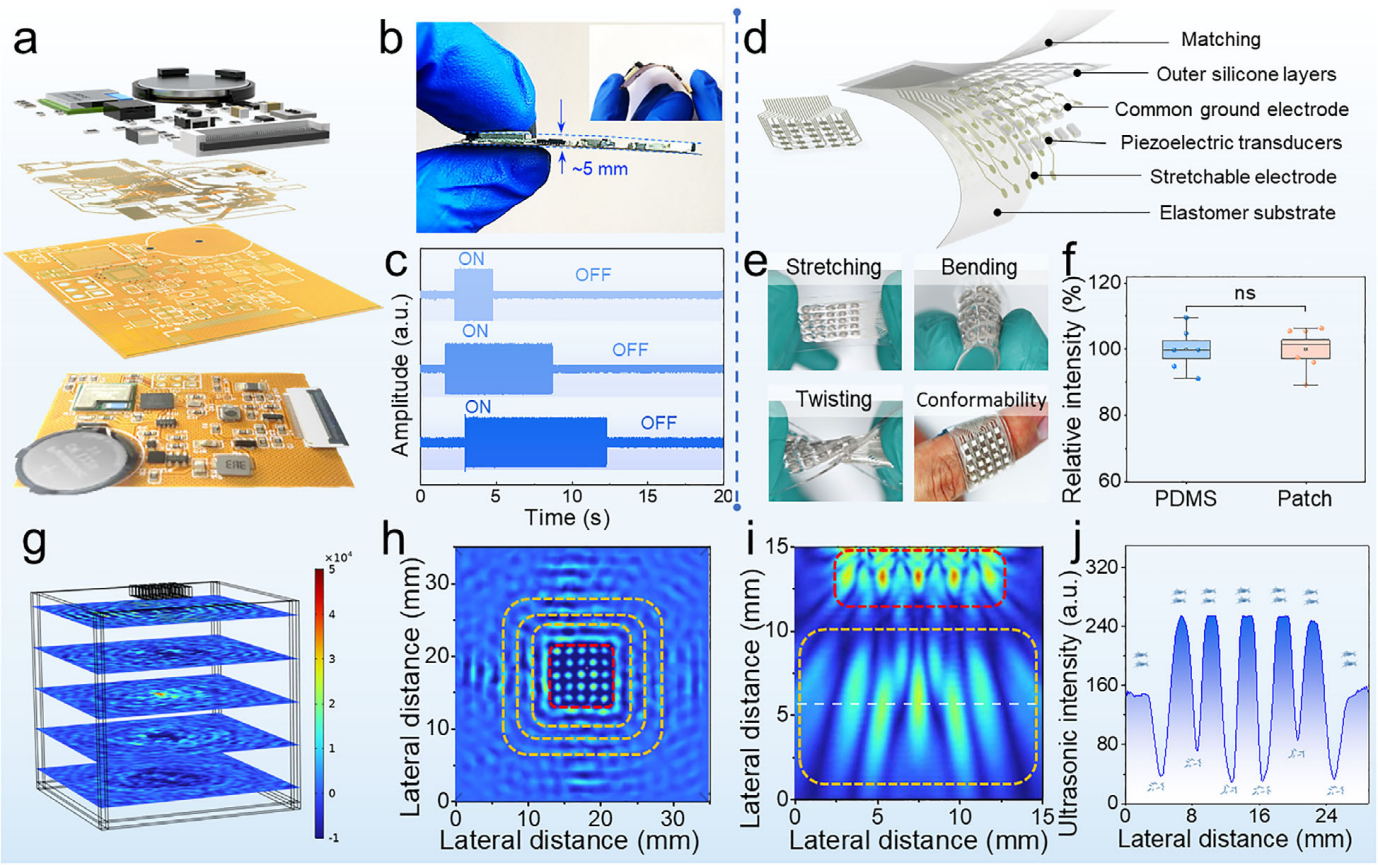
Overview of the fully integrated wearable ultrasound system. (a) Schematic of the exploded view of the circuit board system; The top panel shows the whole device components, including compact battery, fPCB and chip, the middle insets show the main circuit structure diagram, the bottom inset shows the image of the entire circuit board; (b) Side‐view image of the entire circuit board, and the circuit board that bends up and down; (c) Ultrasonic system drives flexible start stop; (d) Layered structure schematic diagram of iWUS Patch; (e) The flexibility of ultrasonic patch (stretching, bending, twisting and conformability); (f) Relative tensile strength of iWUS Patch and PDMS; (g) three‐dimensional‐ultrasound simulation image of the wearable system; Simulation images of ultrasonic energy distribution in cross‐section (h) and longitudinal section (i), and (j) the ultrasonic intensity distribution curve (at the dashed line in image (i), Error bars represent the SD (*P < 0.05, ns not significant, *n* ≥ 3). (Created with Origin).

### 2.3 In Vitro Biological Performance Evaluation of iWUS

As a wearable ultrasound system for wound care, antibacterial performance is an essential core consideration. This study systematically evaluated its sustainable antibacterial performance, as shown in Figure 3. Figure 3a shows a schematic diagram of the antibacterial process of the wearable system, which is expected to achieve effective antibacterial management of wounds through the orderly stimulation of antibacterial substances (PHMB) by ultrasound. We conducted systematic experiments and performance evaluations on the flexibility and controllability of wearable ultrasound systems (Figure 3b). The experimental results show that the system has excellent real‐time response capability, can quickly start and stop ultrasound output, has low operation delay, high stability, and can withstand multiple repeated uses without performance degradation. This performance ensures the reliable operation of the system in dynamic scenarios, demonstrating good consistency and response accuracy during repeated operations, and is suitable for continuous monitoring and treatment scenarios requiring precise control. Through ultrasonic stimulation for the controlled release of PHMB, we characterized the concentration of the released antibacterial agent (Figure 3c and Figure S2). This concentration is a critical parameter influencing both antibacterial efficacy and wound management outcomes, as it directly determines therapeutic efficacy and biocompatibility. The results demonstrate that the system exhibits a high degree of stability and predictability in regulating PHMB release behavior via ultrasound stimulation, thereby providing a robust foundation for efficient and safe antibacterial therapy. Furthermore, we investigated the release behavior of PHMB under continuous ultrasound stimulation at a frequency of once daily. The results showed that during repeated stimulation for more than one week, the released concentration of PHMB exhibited a slow and steady downward trend, with no sharp decrease or sudden decay observed (Figure 3d). This result indicates that the system has good long‐term stability and can continuously and controllably release antibacterial components, providing reliable and long‐lasting antibacterial protection for wound management. Finally, we systematically evaluated the antibacterial performance of the ultrasound‐triggered PHMB release from this system against Escherichia coli and Staphylococcus aureus. The results demonstrate that the released PHMB exhibited significant antibacterial activity, showing potent and sustained inhibitory effects against both common pathogens. Besides, the system achieved notable bactericidal outcomes, in reducing bacterial viability (Figure 3e,f and Figure S3). Scanning electron microscopy (SEM) analysis in Figure S4 confirms that PHMB disrupts bacterial membrane integrity, causing distinct morphological damage and resultant cell death. These findings provide direct visual evidence of its potent bactericidal mechanism and highlight the promising translational potential of this system for advanced antibacterial wound dressings and infection control, laying a robust foundation for its future biomedical development [49].

**FIGURE 3:**
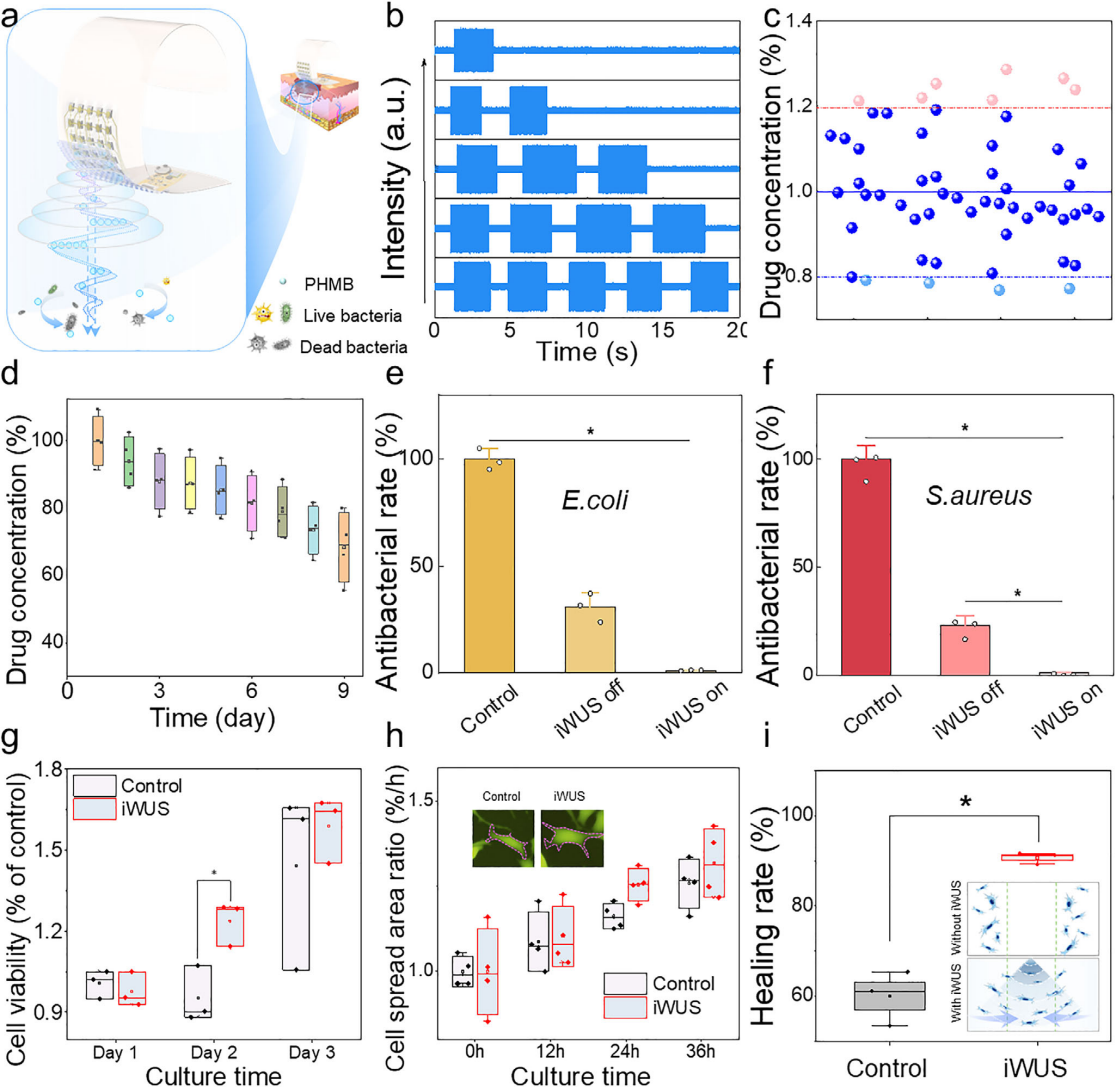
Programmed drug release antibacterial performance and two dimensional wound healing test in vitro of the ultrasound system. (a) Schematic diagram of antibacterial substance release driven by ultrasound from the system; (b) Flexible handling of ultrasonic drive; (c) Stability of ultrasound induced programmed drug release; (d) The attenuation of antibacterial substance release after several days of ultrasonic driving; (e) and (f) The antibacterial stability and durability of system (*S. aureus* and *E. coli*) (*n* = 3); (g) Cell activity under ultrasound; (h) Cell spreading rate under ultrasound. The differences among groups were calculated using one‐way ANOVA; (i) Two dimensional wound healing rate. (The differences among groups were calculated using one‐way ANOVA. *p < 0.05, **p < 0.01.) (Created with Origin).

### 2.4 The Evaluation of Wound Healing Promotion In Vitro

Encouraged by the aforementioned significant in vitro antibacterial effects, we proceeded to assess the cytocompatibility of the system. Representative microscopy images of cells following ultrasonic stimulation are presented in Figure S5, revealing no indication of induced apoptosis. Notably, the ultrasonic treatment did not compromise cell health; rather, it enhanced cellular viability (Figure 3g), showing a significant increase in proliferative activity (Figure S6), and a greater proportion of cells exhibiting well‐spread and adhered morphologies (Figure 3h). The spreading morphology of cells during migration serves as an important indicator of stem cell activation. These data collectively confirm the favorable biosafety profile of the system and underscore its intrinsic potential to facilitate the wound healing process. To systematically evaluate its pro‐healing potential, we next investigated the ability of the iWUS system to promote cell migration and wound closure—using an in vitro scratch assay. Representative images capturing the migratory fronts of cells, with or without iWUS stimulation, are shown in Figure S7. Quantitative analysis confirmed that while wound closure occurred in both groups over time, the healing progress was markedly and significantly accelerated in the iWUS‐treated cohort compared to the untreated control (Figure S8). A schematic illustration proposing a plausible mechanism to explain this enhanced healing response is provided in Figure 3i. During the in vitro experiments, the process by which L929 fibroblasts migrate from high‐energy regions to low‐energy regions under the ultrasound energy field is dynamic (as shown in Figure 2i,j), ultimately manifesting at the macroscopic level as directional migration and accelerated wound closure. Taken together, these findings robustly demonstrate the system's excellent efficacy in promoting cellular repair and its promising translational prospects for medical applications.

### 2.5 Evaluation of the Therapeutic Efficacy of iWUS in Chronic Wound Healing In Vivo

This wearable ultrasound system constitutes an intelligent therapeutic solution for chronic wound management (Figure 4a). By integrating on‐demand treatment modalities, it enables the delivery of precise, adaptive care tailored to the dynamic state of the wound. The design philosophy places a strong emphasis on user‐centricity, incorporating a lightweight and flexible architecture that ensures high practicality, ease of wear, and straightforward operation. Furthermore, the system's low‐power, wireless configuration supports extended, continuous operation. This design critically minimizes disruption to patients' daily activities and mobility, thereby facilitating the preservation of normal life rhythms throughout the treatment period. As a wearable device designed for continuous on‐skin operation, this system exhibits excellent thermal safety. The temperature changes after 1–3 min of on‐skin running are shown in Figure S9; the negligible temperature variation indicates that its use on the human body surface is safe and reliable. Furthermore, its demonstrated skin biocompatibility and reliable attachment capability suggest suitability for long‐term, personalized use in diverse clinical and home‐care settings, as the digital image attached to its skin surface is shown in Figure 4b.

**FIGURE 4:**
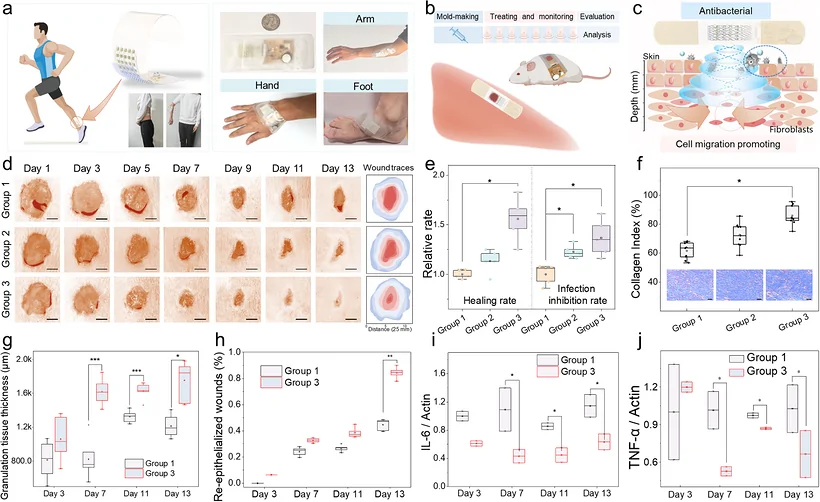
In vivo study of wearable ultrasound system. (a) Schematic diagram of iWUS application on chronic wounds, and digital images adapted to multiple scenarios; (b) Schematic diagram of animal experiment implementation process; (c) Schematic diagram of iWUS promoting wound healing mechanism; (d) Wound contour and infected area during treatment (marked in a darker red to improve visualization) and the wound area traces; (e) Healing rate and infection inhibition rate of wounds at different stages; (f) Masson staining map and relative ratio of tissues in the healing area; (g) Granulation tissue thickness of newly formed skin; (h) Re‐epithelialized wound ratio; IL‐6 concentration (i) and TNF‐a concentration (j) during wound healing. The differences among groups were calculated using one‐way ANOVA. *p < 0.05, **p < 0.01.

To evaluate the practical applicability of the wearable system for wound management, we established a full‐thickness skin defect model in Sprague‐Dawley (SD) rats and subsequently assessed its in vivo therapeutic efficacy (experimental procedure and results are shown in Figure 4b,d). All animal experiments and study protocols were conducted in strict compliance with the National Regulation of China for Care and Use of Laboratory Animals and were approved by the Animal Ethics and Welfare Committee of Shenzhen University (Protocol No. IACUC‐202400008). Building on our previous in vitro experiments on ultrasound‐promoted cell migration and PHMB release, we comprehensively evaluated the performance of the wearable ultrasound device in enhancing wound closure and tissue regeneration in a biologically relevant environment, with the therapeutic process illustrated in Figure 4c. These studies help elucidate its mechanism of action, thereby supporting its potential translation to clinical applications. Chronic diabetic wounds, owing to their intrinsic pathophysiological characteristics, not only exhibit a high propensity for impaired healing but are also highly susceptible to complications such as bacterial infection and suppuration [50]. These complications can severely disrupt the normal wound healing cascade, leading to delayed tissue repair, protracted healing timelines, abnormal or incomplete scab formation, and even frequent infection, thereby further complicating the clinical presentation [14]. Consequently, this study not only monitored the contour of the wound, but also monitored the abnormal healing situation (wound infection area) during the treatment process, as shown in the deep red area in Figure 4d, in which also shown the representative wound morphological changes.

As treatment progressed, the experimental group exhibited a marked and rapid reduction in wound area, with both the rate and efficacy of healing significantly surpassing those of the control group. Concurrently, the infection zone surrounding the experimental wounds was effectively contained, showing no further expansion, which demonstrated a robust local anti‐infective capacity. Collectively, these data indicate that the therapeutic regimen not only effectively accelerates wound closure but also, by inhibiting the proliferation of pathogenic microorganisms, fosters a stable, low‐inflammatory microenvironment favorable for tissue repair. Within this conducive milieu, critical healing processes—including cellular proliferation, migration, and matrix remodeling—proceed in an orderly and coordinated manner. In summary, these results indicate that the treatment not only promoted wound closure but also established a stable and conducive microenvironment for tissue regeneration by suppressing infection, thereby facilitating an orderly and well‐coordinated healing process. As shown in Figure 4e, the wound healing rate and infection inhibition rate were evaluated. The experimental group treated with the integrated wearable ultrasound system (iWUS) exhibited an approximately 50% faster wound healing rate compared to the untreated group. Meanwhile, the reduction in wound infection area in the iWUS group was significantly greater than that in the other control groups, with statistically significant differences.

To gain deeper insight into the biological process of wound healing, we conducted a systematic histological evaluation of wound samples collected at different time points. Using optical micrographs of hematoxylin and eosin (H&E)‐stained sections, we performed a detailed comparative analysis of the histomorphological characteristics among different experimental groups (Figure S10). On day 3, all groups exhibited noticeable inflammatory responses and macrophage accumulation; however, the inflammatory cell density in Group 3 (iWUS group) was significantly lower than that in Group 1 and Group 2, suggesting that this treatment may effectively alleviate the early inflammatory response. Furthermore, fibroblast migration into the wound area was observed in Group 3 as early as day 3, becoming more pronounced on days 9 and 12, which indicates an earlier initiation of the repair process. Although mild inflammation was still present in Group 3 on day 3, inflammatory cells had largely subsided by day 12. In contrast, Group 2 still showed mild inflammatory cell infiltration on day 12, implying a delayed resolution of inflammation. These results provide further histological evidence that the wearable ultrasound treatment modulate the wound microenvironment and repair progression distinctly. Collagen deposition and the regularity of its arrangement are key indicators for assessing the quality of wound healing and the restoration of skin barrier function. As shown in Figure 4f, the experimental group (Group 3) exhibited superior collagen deposition and more organized collagen fiber alignment compared to the other groups, particularly Group 1. These results demonstrate that iWUS treatment not only accelerates wound closure but also effectively promotes collagen synthesis and orderly deposition, thereby significantly improving healing quality, reducing scar formation, and facilitating functional skin recovery.

Furthermore, at different stages of the healing process, the experimental group exhibited greater granulation tissue thickness and a higher re‐epithelialization rate in the newly formed skin compared to other groups (Figure 4g,h). Granulation tissue thickness and re‐epithelialization rate are crucial indicators representing the regeneration of the dermal and epidermal layers, respectively. Adequate granulation thickness reflects robust neovascularization, fibroblast proliferation, and extracellular matrix deposition, providing structural support and nutritional supply for re‐epithelialization. The re‐epithelialization rate, in turn, indicates the speed and completeness of epidermal renewal, which is essential for restoring the skin barrier function [51, 52]. Together, these parameters serve as core metrics for evaluating the efficiency and quality of wound healing. It is well established that inflammatory levels at the wound bed serve as a key indicator of the microenvironmental status during chronic wound treatment. In this study, we systematically measured the inflammatory markers IL‐6 and TNF‐α in wound tissues throughout the treatment period to objectively evaluate the degree of inflammation at the lesion site, as shown in Figure 4i,j. At various time points, the concentrations of IL‐6 and TNF‐α in the treatment group (Group 3) were significantly lower than those in the other groups, particularly compared to the blank control group (Group 1). These results indicate that the therapeutic strategy effectively suppresses excessive inflammatory responses and alleviates the inflammatory microenvironment, thereby creating favorable conditions for orderly wound repair and tissue regeneration.

In summary, the iWUS system effectively enhances directed cell migration to the wound site, facilitating granulation tissue formation and epithelial regeneration, which collectively contribute to a shortened healing timeline. This work provides vital theoretical and experimental evidence supporting the application of wearable ultrasound technology for the management of chronic and refractory wounds.

### 2.6 Underlying Wound Healing Promoting Mechanism of iWUS System

To evaluate the effects of iWUS treatment on the wound microenvironment [53], RNA sequencing (RNA‐seq) was performed on wound tissues collected 7 days post‐treatment, with wound tissues from the blank group serving as controls. Differential expression analysis using edgeR (Figure 5a) revealed 1,317 upregulated and 1,216 downregulated genes in the iWUS group (Group 3). Enrichment analysis was subsequently conducted to explore the gene expression profiles associated with inflammation and wound healing [54]. Hierarchical cluster analysis revealed a downregulation of inflammatory genes related to inflammation and an upregulation of genes associated with wound healing after the iWUS treatment (Figure 5b,c). Specifically, the downregulation of interferon‐induced innate immune response genes (e.g., Gbp1, Tarm1, Bpi) indicates a transition of wound inflammation into the resolution phase, representing a pro‐healing signal [55, 56, 57]. Concurrently, the upregulation of genes associated with anti‐inflammatory responses, tissue protection, and repair initiation (e.g., Serpina3n, Defa5, Mc2r) suggests a favorable trajectory in wound healing (Figure 5b) [58, 59]. The downregulation of genes associated with fibrotic tendency, oxidative stress, and epithelial dysfunction (e.g., Dao and Slco1c1) indicates that the wound has fully transitioned from a non‐healing, stalled state into an orderly repair phase [60, 61, 62]. Meanwhile, the expression of genes involved in re‐epithelialization, angiogenesis, and matrix remodeling (e.g., Cdh19, Krt13, and Ereg) suggests that the wound has moved beyond the inflammatory stagnation phase and entered an active remodeling stage, serving as a robust hallmark of pro‐healing progression (Figure 5c) [63, 64, 65].

**FIGURE 5:**
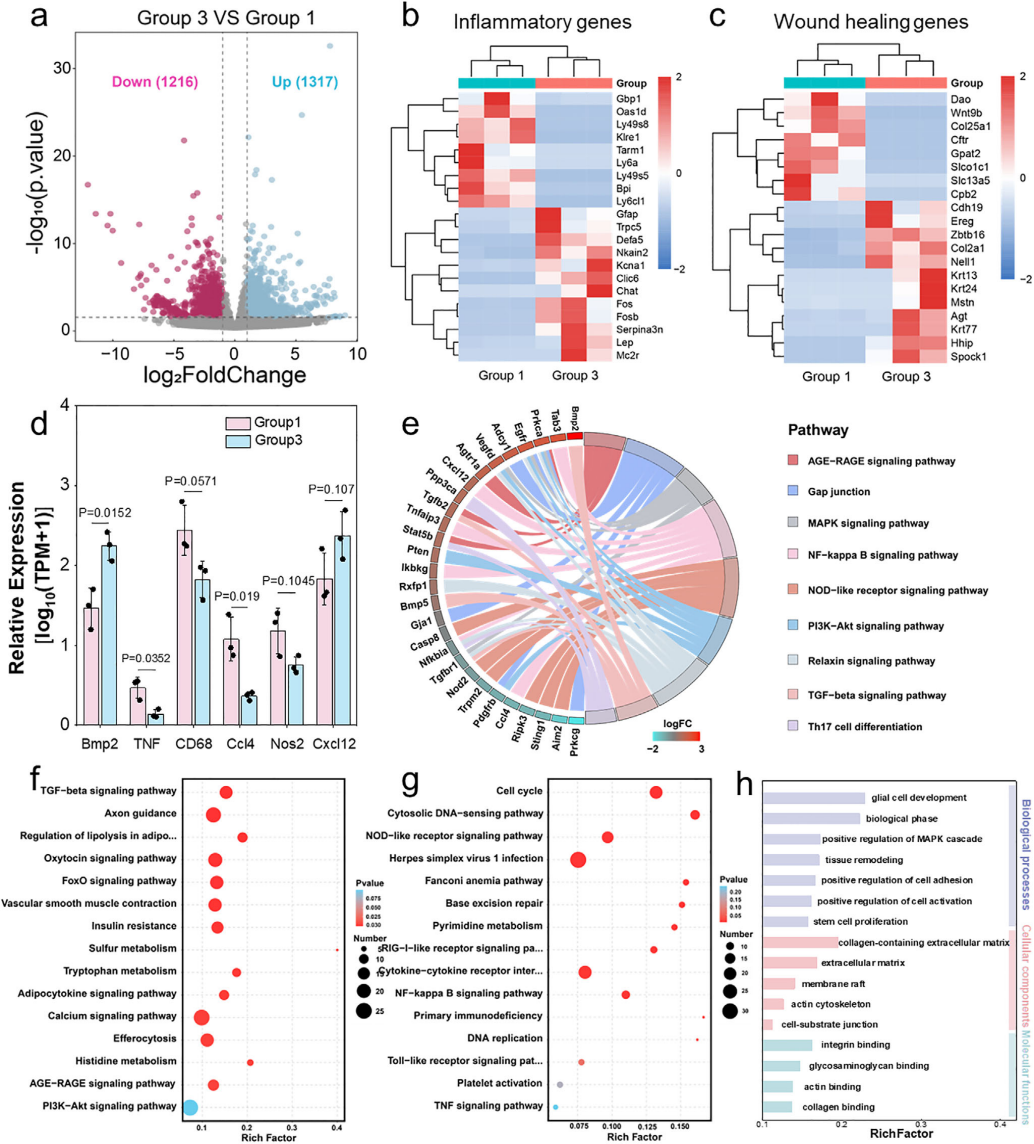
Analysis of DEGs in wound tissues after iWUS treatment. (a) Volcanoplots showing the upregulated and downregulated genes in the iWUS group (compare with group 1); (b) and (c) showing the heat maps of inflammation genes and wound healing genes; (d) Relative expression of genes associated with wound regeneration (including inflammation and wound healing); (e) Gene chord plot revealed that the regulation of related genes in different clusters; (f) and (g) KEGG‐enriched bubble maps of upregulated and downregulated DEGs, with larger bubbles indicating more pathway DEGs; (h) GO enrichment analysis of molecular functions, cellular components, and biological processes of genes. (Created with R).

Upregulation of *Bmp2*, a bridging molecule between ultrasound physical stimulation and cell recruitment (compared with Group 1), indicates that ultrasound stimulation exerts a beneficial effect on chronic wounds in the iWUS group (Figure 5) [66, 67, 68]. The concomitant downregulation of pro‐inflammatory genes, including *TNF*, *CD68*, *Ccl4*, and *Nos2*, further suggests that ultrasound likely plays a critical role during the treatment process [54, 56, 69, 70]. Gene Ontology (GO) and chord diagram analysis revealed that iWUS‐regulated genes were mainly enriched in different gene sets associated with immune modulation, cell proliferation, and tissue repair processes (Figure 5e) [12]. Notably, ultrasound stimulation combined with programmed antimicrobial activity of PHMB (Group 3) led to upregulation of Bmp2, a gene responsive to ultrasound mechanical stimulation and involved in promoting angiogenesis as well as regulating neuro‐immune repair [66, 67]. Furthermore, significant upregulation of Egfr, and Vegfd in the iWUS group compared with the control group indicates that this therapeutic strategy effectively activates tissue repair mechanisms, including enhanced cell migration and angiogenesis [12, 71, 72, 73]. Activation of pathways such as the TGF‐β signaling pathway and MAPK signaling pathway, along with suppression of the NOD‐like receptor signaling pathway, suggests that iWUS treatment promotes healing of infected chronic wounds through ultrasound‐mediated modulation of these pathways [74, 75].

Furthermore, Kyoto Encyclopedia of Genes and Genomes (KEGG) was used to analyse the potential signaling pathways. The findings demonstrated that iWUS treatment enhanced signal transduction through the PI3K‐Akt signaling pathway, which is associated with ultrasound‐mediated activation of cells and proteins (Figure 5f) [74]. In contrast, M1 polarization‐associated inflammatory pathways, including the NF‐κB and TNF signaling pathways, were downregulated (Figure 5g). According to related studies, this change is beneficial for improving the microenvironment, facilitating the wound‐healing process to proceed in an orderly manner, and may even ameliorate scar formation [13, 76]. Collectively, these results indicate that iWUS treatment accelerates wound healing by suppressing inflammatory response pathways while promoting tissue repair and angiogenesis. Gene ontology (GO) enrichment analysis (Figure 5h) provided insights into the different biological processes, cellular components, and molecular functions associated with the DEGs between group 3 and group 1. Kyoto Encyclopedia of Genes and Genomes (KEGG) analysis predicted the potential signaling pathways [12]. The findings revealed that the iWUS treatment enhanced the signal regulation of MAPK pathways, which are associated with M2 macrophage activation (Figures 5e and 1e).

## 3 Conclusions

In this study, we developed a flexible and convenient wearable ultrasound system for the treatment and management of chronic wounds. The system consists of a highly integrated ultrasound driving circuit, a drug‐loaded ultrasound patch, and a remotely controllable terminal, offering excellent connectivity and ease of operation. It conforms closely to body surfaces with varying curvatures, adapts to multiple wound types, and supports remote control of ultrasound activation and termination via a terminal, providing a portable solution for on‐demand medical applications. By activating the release of an antibacterial agent (PHMB) through ultrasound stimulation, we demonstrated the system's controllable antibacterial functionality and sustained antimicrobial efficacy. After achieving promising results in an in vitro two‐dimensional healing model, we further validated the system's effectiveness in a rat full‐thickness skin defect model. The results indicated that the iWUS system not only significantly accelerates wound healing and controls infection effectively but also improves collagen alignment and increases dermal thickness in the newly formed skin. These findings confirm its dual capability to promote both structural and functional skin regeneration while enhancing barrier recovery. In summary, the wearable ultrasound system developed in this study enables effective and intelligent wound management, offering a novel approach to chronic wound therapy and providing an important strategic pathway for the development of wearable medical devices.

## Author Contributions

**Faxing Zou**: investigation, Writing – original draft, project administration, conceptualization, methodology. **Yansen Wang**: methodology. **Yong Luo**: methodology. **Wenxuan Zhuang**: methodology. **Mingyu Zhu**: methodology, investigation. **Min Long**: writing – review and editing, funding acquisition. **Qingzhou Liu**: writing – review and editing, formal analysis. **Tailin Xu**: supervision, writing – review and editing, funding acquisition.

## Funding

This work was supported by National Natural Science Foundation of China (62501404, 32671868, 62301325, 22427806), Shenzhen Medical Research Fund (D2601007), Key Project of Scientific and Technological Breakthroughs in the Field of Life and Health in Chongqing Shapingba District (20240101), Shenzhen Science and Technology Program (JCYJ20240813142503006), Shenzhen Overseas Talent Program.

## Conflicts of Interest

The authors declare no conflicts of interest.

## Supporting information




**Supporting File**: advs78619‐sup‐0001‐SuppMat.docx.

## Data Availability

The data that support the findings of this study are available from the corresponding author upon reasonable request.
