# Supplementary material for: Fully Integrated Wearable Ultrasound System for Continuous Promotion of Wound Healing
Source: Adv Sci (Weinh). 2026 Nov 9:e78619. Online ahead of print. doi: 10.1002/advs.78619 (PMC13749011; doi:10.1002/advs.78619)
Supplement: Supplementary file 1 [file ADVS-9999-e78619-s001.docx]

Supporting Information

Fully Integrated Wearable Ultrasound System for Continuous Promotion of Wound Healing

*Faxing Zou, Yansen Wang, Yong Luo, Wenxuan Zhuang, Mingyu Zhu, Min Long*, Qingzhou Liu*****,*** *Tailin Xu**

F. Zou

School of Life and Health Technology, Dongguan University of Technology, Dongguan, Guangdong, China.

F. Zou, Y. Luo, W. Zhuang and T. Xu

The Institute for Advanced Study (IAS), Shenzhen University, Shenzhen, Guangdong 518060, People’s Republic of China.

E-mail: [xutailin@szu.edu.cn](mailto:xutailin@szu.edu.cn) (T. Xu)

Y. Wang

Department of Medical and Health Materials, SINOPEC Beijing Research Institute of Chemical Industry Beijing 100013, P. R. China

M. Zhu and M. Long

Department of Endocrinology, The First Affiliated Hospital (Southwest Hospital) of Army Medical University, Chongqing, 400038, China

Q. Liu

Guangdong Laboratory of Artificial Intelligence and Digital Economy (SZ), Shenzhen 518060, China

**Keywords:** wearable ultrasound, chronic wound, antibacterial, promote healing.

Experimental Section

*Materials**:* Cu foil (50 μm thick, MicroThin) was produced by Mitsui Mining & Smelting Co., Ltd. The polydimethylsiloxane (PDMS) and the corresponding curing agent were purchased from Dow Europe GmbH. 1-3 composite (PZT-5) was purchased from Del Piezo Specialties. Polyhexamethylene biguanidine hydrochloride (PHMB) was obtained from Shanghai Macklin Biochemical Co., Ltd. Staphylococcus aureus (S. aureus ATCC 25922) and Escherichia coli (E. coli, DH10B) were collected from China General Microbiological Culture Collection Center) and cultured with Lennox Broth (LB, Sangon Biotech) and agar (Macklin). Hoechst 33342 were supplied by Leagene Biotech Co., Ltd. (Beijing, China). Phosphate Buffered Saline (PBS) and L929 cell were obtained from Wuhan Servicebio Technology Co., Ltd. Dulbecco’s modified Eagle’s medium (DMEM), trypsin-EDTA and Penicillin-streptomycin (PS) were purchased from Gibco Life Technologies (AG, Switzerland). 4% Paraformaldehyde fix solution was obtained from Beyotime. The Calcein AM were supplied by Shanghai yaji biotechnology Co., Ltd. (Shanghai, China).

*Fabrication of the iWUS**:* The fabrication of the conformal circuit of the iWUS patch is outlined in Figure 1a. First, wet etching and photolithography were applied to both side of a laminated film consisting of Cu with a thickness of 50μm, respectively. Here, the circuit design of a thin Cu layer is of great significance to ensure the electrical performance of the iWUS patch. The both sides covered with a layer of polydimethylsiloxane (PDMS) is beneficial to increasing the ductility of Cu by delocalizing its deformation, enhancing the restoring ability and antifatigue ability. A PZT plate was polarized and mechanically cut into 2.0 mm × 2.0 mm × 1.0 mm thin slices. Following, the fabricated piezoelectric 1-3 composite element are arranged according to the design form, assembled with PDMS and conductive silver paste (etc.) to form small, flexible, and highly conductive ultrasonic patch, with reserved wiring interfaces. Then, the PCB substrate for assembling the ultrasonic driver was designed by design software and manufactured by a commercial service.

*Characterization of the iWUS**:* The iWUS driving system and ultrasound patch responsiveness were investigated through repeated on/off cycling operations, with the specific ultrasound signals detected and recorded by the oscilloscope. To assess the ultrasound-driven drug release performance, commercial dressings were first immersed in a 0.02% PHMB solution for 24 hours, followed by daily one-minute actuation using the ultrasound system developed in this study; the amount of drug released was subsequently collected and quantified.

*Finite element analysis (FEA):* FEA simulations were carried out to simulate the acoustic field distributions of the US transmitters and the corresponding US-induced piezo-potential distributions in elements, respectively. In acoustic field simulations of a 2D array transducer, a backpropagation method, angular spectrum method, was used to determine the amplitude and phase distribution on the 2D array for either letter. In simulations, the physical domains considered include electrostatics, pressure acoustics (frequency-domain), and solid mechanics, as well as the coupled interfaces of the piezoelectric effect and acoustic-structural boundary. Material parameters were set according to supplier data sheets and experimental measurements. The geometries of the components were strictly in accordance with the designs.

*In vitro antibacterial activity:* After assembling the ultrasound system with a dressing loaded with PHMB, the solution containing the released PHMB drug was stimulated by the ultrasound system at different times, and then the concentration of PHMB in the tube was measured, subsequently. The absorbance of the solutions was measured using the ultraviolet spectrophotometer (HP 8453, USA) at the wavelength of 235 nm. The concentration of PHMB in test tubes were determined from the comparison of the absorbance with PHMB standard curve, ultimately calculate the drug release after ultrasound stimulation. In addition, Luria-Bertani (LB) liquid medium was used to culture the high activity bacteria (E. coli or S. aureus) for 12 h, and then the bacteria were diluted to 10^5^–10^6^ CFU/ ml. Add the solution released by PHMB to the diluted bacterial solution. After different treatments, the treatment bacterial fluid was evenly poured on the solid AGAR mediums, culturing in constant temperature and humidity incubator for 24 h, the number of bacterial colonies in mediums were measured and recorded. To further assess the antibacterial effect of the samples, the morphology of bacteria incubated on the samples was conducted. After fixed with 4 % paraformaldehyde solution, the samples with bacteria were dehydrated with gradient concentration of ethanol and lyophilized. Finally, the samples were observed by SEM after sprayed gold.

*In vitro biocompatibility:* NCTC clone 929 (L929): Fibroblasts were maintained in Dulbecco's Modified Eagle Medium (DMEM) supplemented with 10% fetal bovine serum (FBS), 1% L-glutamine, and 1% antibiotic-antimycotic, at 37°C in a humidified atmosphere of 5% CO₂. When cells reached approximately 80% confluence, adherent fibroblasts were detached using 0.05% trypsin-EDTA (GIBCO) and subsequently passaged. L929 cells were seeded in a 48-well plate and cultured to approximately 70% confluence over 48 hours. A single linear scratch wound was then created in each well using a 200 µL pipette tip. The cells were subsequently subjected to stimulation from an iWUS system for 3 minutes every 12 hours. Following stimulation for 0, 12, 24, and 36 hours, the culture medium was removed, and each well was gently rinsed three times with phosphate-buffered saline (PBS) to remove cellular debris. The cells remaining in the well were stained using a LIVE/DEAD Viability/Cytotoxicity Kit according to the manufacturer's instructions. Fluorescent images were captured using an inverted microscope (PUDA FM-600C, China). In addition, the contour and relative area of the cells were measured and analyzed using the software (ImageJ).

*Wound healing assays**:* All animal experiments and study protocols were conducted in strict compliance with the National Regulation of China for Care and Use of Laboratory Animals and were approved by the Animal Ethics and Welfare Committee of Shenzhen University (Protocol No. IACUC-202400008). A streptozotocin (STZ)-induced diabetic rat model was established using male Sprague-Dawley (SD) rats (Beijing Viton Lihua Laboratory Animal Technology Co., Ltd.), housed individually. STZ, a known agent for inducing type I diabetes mellitus, was administered as a single intraperitoneal injection at 65 mg/kg body weight after a 14-hour fast. The compound was prepared as a 1% solution in 0.1 mM/L citrate buffer (pH 4.2–4.5). Diabetes was confirmed by measuring random blood glucose levels from the tail tip at 72 hours and on day 7 post-injection using a glucometer; a level of ≥11.1 mM/L was required for successful induction, with a second STZ injection given if this criterion was not met. Following model confirmation, rats were monitored daily via body weight measurement and tail vein blood collection. For wound creation, successfully modeled rats were anesthetized with isoflurane, and a circular full-thickness wound of approximately 1 cm in diameter was created on the shaved dorsal skin using a sterile biopsy punch. Subsequently, 15 µL of phosphate-buffered saline (PBS) containing 105/ml concentration of S. aureus was injected into the wound bed (once a day for 5 days to ensure wound infection), and the wound was covered with a 3M dressing to prepare for the experiment.

The rats were randomly divided into three groups. In the group 1, the wounds of rats were only treated with commercial dressings. In the group 2, the wounds were treated with iWUS system which the transducer was affixed to the commercial 3M dressing loaded with antibacterial drug PHMB (but without ultrasound controlled release). In the ultrasound experiment (Group 3), the transducer was affixed to the commercial 3M dressing loaded with antibacterial drug PHMB, with the therapeutic ultrasound parameters including a frequency of 1 MHz, an electrical output power of 5.4 W, a sound intensity of 1.7 W/cm², a duty cycle of 50%, a pulse repetition frequency of 1.0 MHz, and a pulse length of 5 × 10⁻⁷ s. Ultrasound treatment was administered once a day for 3 minutes. The treatment process spanned 13 days, and wound closure was monitored by capturing photographs at different intervals. The area of the wound was measured using the ImageJ software. The rat was euthanized from each group periodically. The entire 1*1 cm area of skin, including the wound site, was removed. The tissue from the wound was fixed with paraformaldehyde and embedded in paraffin for histological processing. The embedded tissue was cut into 6 μm thick sections, mounted on glass slides, and the slice was stained with Masson stain and scanned.

*Transcriptome analysis:* The S. aureus-infected wound tissues from SD rats were collected on day 7 and washed with saline. And then, the total RNA was extracted from tissues using TRIzol™ Reagent. RNA quality was assessed with a NanoDrop instrument, and RNA concentration was measured using the Qubit RNA BR (Broad-Range) Assay Kit. RNA purification, reverse transcription, and library construction were performed using the Hieff NGS® EvoMax RNA Library Prep Kit (dNTP). Sequencing was carried out on the BGI DNBSEQ-T7RS platform with the corresponding FCL PE150 V3.0 kit. To identify differentially expressed genes (DEGs) between two samples, transcript expression levels were quantified using the transcripts per million (TPM) method. Gene quantification was conducted with StringTie, and differential gene expression analysis was performed using DESeq2. FDR≤ 0.05 (DESeq2) were significantly different expressed genes. Additionally, functional enrichment analyses were conducted using GO and KEGG to identify DEGs that were significantly enriched in Gene Ontology (GO) terms and metabolic pathways, compared to the whole transcriptome background, with a Bonferroni-corrected P-value ≤ 0.05. GO enrichment analysis was performed using Goatols, while KEGG pathway analysis was carried out using KOBAS (<http://kobas.cbi.pku.edu.cn/home.do>).

*Statistical Analysis:* Data are expressed as mean ± standard deviation (SD). Statistical significance was determined by one-way analysis of variance (ANOVA). A p-value of less than 0.05 was considered statistically significant, with specific levels denoted as follows: *p < 0.05, **p < 0.01.

Supporting Figures

**Figure S1.** Photographs of ultrasound system (driver module and patch).

**Figure S2.** Standard curve of PHMB drug concentration intensity.

**Figure S3.** Plate colony-counting experiments of after using different treatments.

**Figure S4.** Scanning electron microscopy images of bacterial death after ultrasonic treatment.

**Figure S5.** Cell growth status with or without ultrasound system stimulation.

**Figure S6.** Cell proliferation rate under iWUS stimulation or not.

**Figure S7.** Representative images of healing process of L929 under iWUS stimulation at 0, 12, 24, and 36 hours.

**Figure S8.** Cell migration rate stimulated by ultrasound.

**Figure S9.** The temperature changes after 1–3 min of on-skin running, and representative infrared imaging images.

**Figure S10.** HE staining of wound tissues with different treatment days (neutrophils: red arrows, fibroblasts: blue arrows).
